# Influence of Flue Gas Injection on the Long-Term Durability of a Natural Draft Concrete Cooling Tower

**DOI:** 10.3390/ma12132038

**Published:** 2019-06-26

**Authors:** Jianmin Du, Wenjuan Zhuang, Guo Li, Pei Zhang

**Affiliations:** 1 Jiangsu Key Laboratory of Environmental Impact and Structural Safety in Engineering, China University of Mining and Technology, Xuzhou 221116, China; 2 State Key Laboratory for Geo-mechanics and Deep Underground Engineering, China University of Mining and Technology, Xuzhou 221116, China; 3 Jiangsu Collaborative Innovation Center for Building Energy Saving and Construction Technology, Jiangsu Vocational Institute of Architectural Technology, Xuzhou 221116, China

**Keywords:** cooling tower, concrete, flue gas injection, durability monitoring, numerical simulation

## Abstract

The article undertakes the very important topic of the long-term durability of concrete in a natural draft concrete cooling tower with flue gas injection. The corrosive conditions, including temperature, relative humidity, and CO_2_ and SO_2_ gas concentrations, near the inner wall of a cooling tower with flue gas injection were monitored in real time to obtain the long-term durability performance of concrete. The pH and chemical compositions of the condensed liquid that adhered to the tower’s inner face and the macromorphology, compressive strength, and neutralization depth of in situ specimens were tested periodically. In addition, a finite element numerical simulation was conducted to simulate and verify the concentration distributions of CO_2_ and SO_2_ in the flue gas in the cooling tower. The results showed that the cleaned flue gas was enveloped, diluted, and uplifted by hot vapor in the cooling tower, and its concentration decreased. Meanwhile, the effective diffusion radius increased gradually as the flue gas rose. With the same elevation in the cooling tower, the concentration of flue gas decreased rapidly from the central point to the surrounding area. The air near the inner surface of the cooling tower was merely dampened air with a low concentration of acidic gas due to the gigantic diameter of the cooling tower. As a result, the injection of cleaned flue gas will not evidently increase the corrosion risk in a natural draft concrete cooling tower.

## 1. Introduction

Electricity is the basis of daily operations in modern society, and coal-fired power generation remains the most important method to obtain electricity in most countries worldwide [1]. The flue gas produced by fossil fuel burning generally contains large amounts of acid gases, such as SO_x_ and NO_x_, which will cause serious environmental pollution if they are not properly treated [2,3]. Washing flue gas in an alkaline liquid has been the primary technology for cleaning flue gas since the 1970s and 1980s [4,5]. However, this process reduces the temperature of flue gas to 50–60 °C. As a result, dispersing the gas by using a classical smoke stack is difficult. When flue gas is introduced directly into a cooling tower, the massive hot vapor in the cooling tower develops a ring air curtain for the flue gas, and this curtain envelops and uplifts the flue gas. Hence, the height of the smoke plume is increased, and the dilution and diffusion of pollutants in the flue gas are improved. Such a technology is favorable for the cleaned flue gas re-heater and smoke stack and improves the energy efficiency of coal-fired plants [2,3]. However, several disadvantages exist, and the most important among them is the long-term durability of cooling towers [6,7].

The inner surface of the tower wall is attacked chemically by low-concentration acid vapor because residues of SO_x_ and NO_x_ remain in the cleaned flue gas [2,3]. The conventional approach for addressing this potential corrosion risk is to apply acid-resistant coatings on the inner surface of cooling towers [8,9]. However, the effective service lives of polymer coatings, which require continuous repainting to meet service life requirements, are usually short because of aging effects [10,11]. Continuous repainting is time-consuming and laborious, and it results in a substantial increase in maintenance costs due to the gigantic surface area of cooling towers. A type of special acid-resistant high-performance concrete was developed previously to meet the requirement of corrosion protection; however, the performance of this concrete during the power plant service period was not reported, and the preparation of such concrete will also evidently increase the construction cost of a cooling tower [2,4].

The cooling tower is an essential component of coal-fired power plants, and its structural safety and long-term durability have been the focus of researchers’ attention [12,13,14,15,16,17,18]. Considering the short application time of cooling towers with flue gas injection, recent studies concentrated on anti-corrosion design, whereas the real durability performance of cooling towers during power plant operation lacks research attention [1,2,3,4,5]. The objective of the present study is to investigate the corrosive conditions near the inner surface of a cooling tower with flue gas injection and the durability performance of the tower concrete. The findings of this work will provide reliable suggestions for the long-term durability design of cooling towers with flue gas injection.

## 2. Flue Gas Monitoring and Experimental Plan

### 2.1. Project Background

**Figure 1:**
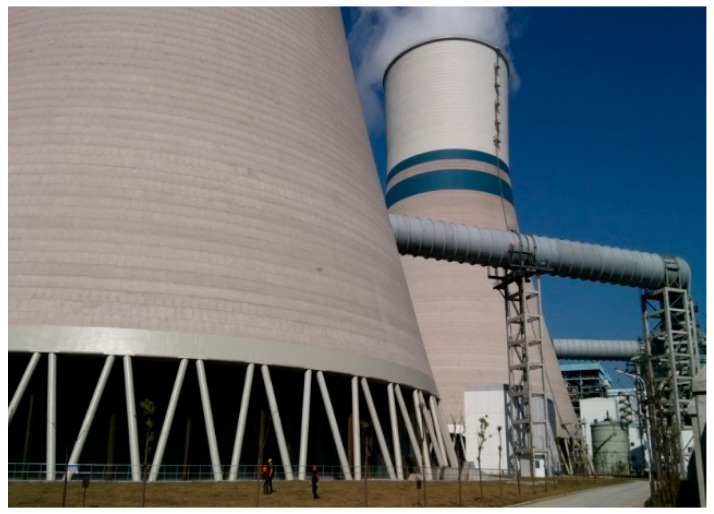
Cooling tower with a flue gas pipe.

A project involving new cooling towers with flue gas injection located in Lu’an Power Station (Lu’an, China) was used as the background. This project is composed of two 600 MW ultra-supercritical coal-fired units, and the cooling system is a secondary circulation water supply system equipped with a counter-current natural draft cooling tower with an area of 8500 m^2^. The cooling tower is 150 m high, and the height of the flue gas inlet is 42 m (Figure 1). The construction of this project began in September 2012, and it was completed and put into operation in March 2014.

### 2.2. Monitoring Plan and Experimental Methods

A special C45 acid-resistant concrete was designed in consideration of the potential risk of acid attack and cooling towers’ expected service life of 30 years. The mixture proportion of the designed acid-resistant concrete is shown in Table 1. The binders in the raw materials of the tower concrete were P·O 42.5 ordinary Portland cement, grade I low-calcium fly ash, and silica fume, with their chemical compositions shown in Table 2. The coarse aggregate was local crushed continuous grading limestone with a particle size of 5–31.5 mm. The fine aggregate was local grading zone II river sand with a fineness modulus of 2.5. The mixing water was ordinary tap water, and the water reducer was a type of polycarboxylate superplasticizer.

**Figure 2:**
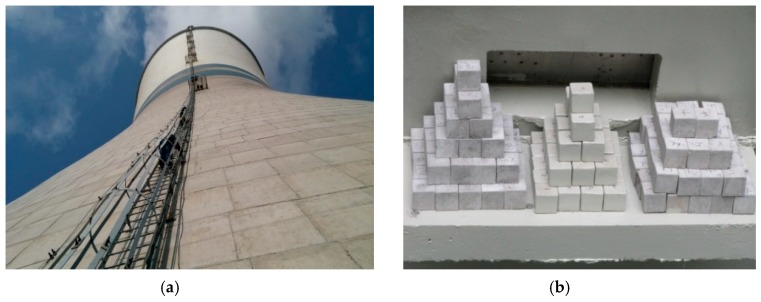
Auxiliary inspection facilities of the cooling tower. (**a**) Steel platform outside the tower; (**b**) concrete platform inside the tower.

Health monitoring is an important means to ensure the safety performance of major civil engineering structures [19,20,21]. Steel and concrete platforms were built at the middle and throat positions outside and inside the tower, respectively, to monitor the real-time development of corrosive conditions in the cooling tower and the tower concrete’s durability performance (Figure 2). Three locations were designed for the placement of in situ concrete specimens and temperature, relative humidity, and CO_2_ and SO_2_ sensors. The detailed monitoring plan is shown in Table 3. The planned monitoring time was two years. The mixture proportion and curing method of the in situ concrete specimens were exactly the same as that of the tower concrete.

Temperature, relative humidity, and CO_2_ and SO_2_ concentration data were collected with a small data acquisition instrument every half an hour and saved automatically. Generally, the condensate is only observed on the inner wall surface when ambient temperature is below the dew point of vapor in a cooling tower; thus, the condensate in this study was sampled twice in the winter season during the entire monitoring period. The pH and chemical composition of the condensate samples were analyzed with a PHS–25 pH meter (Shanghai INESA Scientific Instrument Co., Ltd., Shanghai, China) and ISC-1500 ion chromatograph (Dionex Corporation, Sunnyvale, CA, USA), respectively. In situ concrete specimens were retrieved at the age of 300 and 510 d after power plant operation, and visual appearance, compressive strength, and neutralization depth were observed or tested.

## 3. Results from Monitoring

### 3.1. Temperature and Relative Humidity near the Inner Face of the Cooling Tower

**Figure 3:**
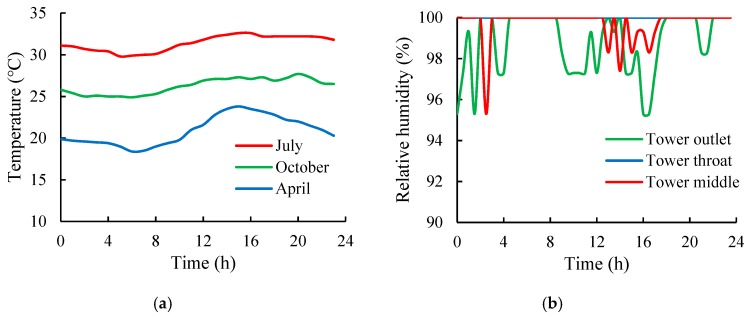
Typical temperature and relative humidity over 24 h in the cooling tower. (**a**) Temperature; (**b**) relative humidity.

According to the measured data, the temperatures near the inner surface of the cooling tower were considerably affected by the external environmental temperature and changed regularly with seasons and day and night cycles due to the thin-shell structure of the cooling tower (the average thickness of the tower wall concrete was 25 cm). Generally, the temperatures near the inner wall surface were slightly higher than that of the outside environment. However, the temperature differences between different locations, such as at the tower outlet, tower throat, and tower middle, were small. The moisture contents in a cooling tower are always high, and the moisture transmission resistance in concrete is also high [22]. Therefore, regardless of the changes in the relative humidity of the external environment outside the cooling tower, the relative humidity near the inner wall surface remained high (basically maintained at above 95%). The typical variations of temperature and relative humidity in a day are presented in Figure 3.

### 3.2. Concentrations of CO_2_ and SO_2_ near the Inner Wall of the Cooling Tower

**Figure 4:**
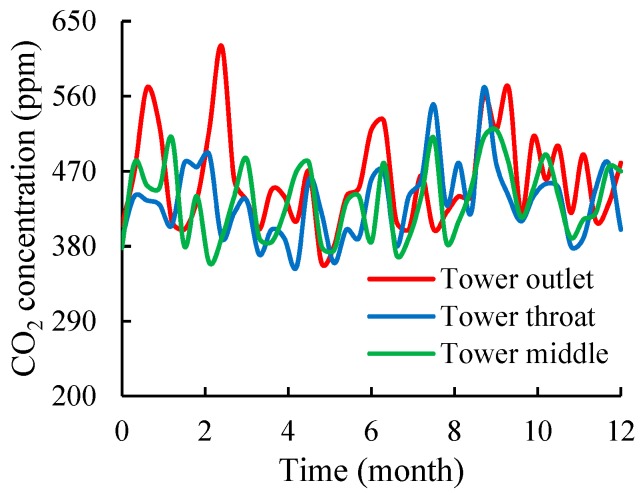
CO_2_ concentration over a year in the cooling tower.

On the basis of the measured data, the development of CO_2_ concentration near the inner surface of the cooling tower over a year is plotted and shown in Figure 4. Although the flue gas emitted by coal-fired plants generally contains a large amount of CO_2_ [2,3], the CO_2_ concentration near the inner surface of the tower showed a low concentration with a range of 350–650 ppm. This value is not considerably different from the CO_2_ concentration of the ambient atmosphere outside the tower. The concentrations of CO_2_ at different positions inside the tower, such as the tower outlet, tower throat, and tower middle, also showed no evident differences. The data obtained for SO_2_ were similar to those for CO_2_ but with a lower concentration of 0–0.5 ppm. The monitoring results of CO_2_ and SO_2_ concentrations indicate that no high concentration of acid gas was present around the inner surface of the cooling tower.

### 3.3. Condensate Attached on the Inner Wall of the Cooling Tower

Table 4 lists the average pH value and anion contents of the condensate obtained. The pH value of the condensate at each location was basically between 6.0 and 6.5, and the closer the location was to the top, the lower the pH value was. However, the condensed liquid was only slightly acidic, and such pH values are inconsistent with the low pH values of 1–2 predicted by several authors [3,4,12]. However, these pH values agree well with the aforementioned results of acid gas content near the inner wall of the cooling tower. Further observation indicated that the contents of corrosive ions in the condensed droplets were generally low. Particularly, the contents of Cl^−^ and SO_4_^2−^ ions were slightly higher than those of F^−^ and NO^3−^ ions. However, such low contents basically indicate no evident corrosion risk in concrete [23]. In sum, the discharge of the cleaned flue gas into the cooling tower did not substantially increase the corrosion risk in the concrete tower wall.

### 3.4. Macromorphology of In Situ Specimens

**Figure 5:**
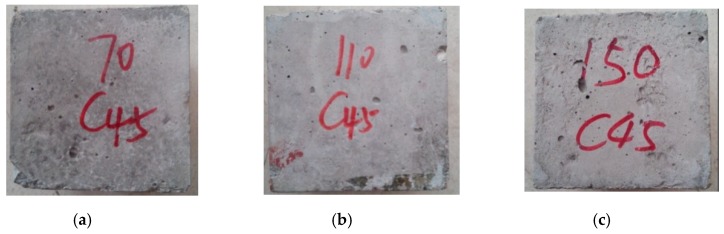

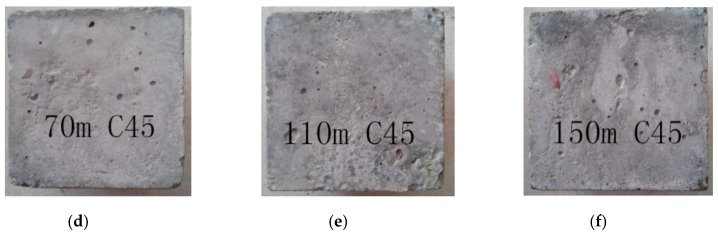
Photographs of specimens placed in different locations in the cooling tower. (**a**) Tower middle (300 d); (**b**) tower throat (300 d); (**c**) tower outlet (300 d); (**d**) tower middle (510 d); (**e**) tower throat (510 d); (**f**) tower outlet (510 d).

Partial specimens were retrieved from the platforms inside the cooling tower, and typical photographs of these specimens are shown in Figure 5. After power plant operation for 300 and 510 days, no sign of corrosion and no evident differences between specimens at different locations were observed on the specimens’ surfaces; moreover, no holes that might be due to acid attack [24] nor white crystals caused by sulfate attack [23] were found.

### 3.5. Compressive Strength and Neutralization Depth of in Situ Specimens

**Figure 6:**
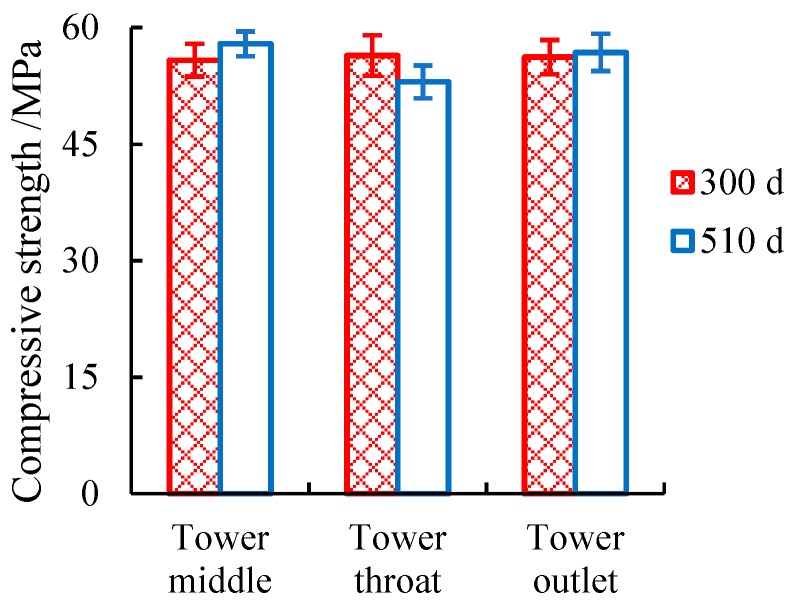
Compressive strengths of in situ specimens at different locations.

The compressive strengths of the in situ concrete specimens are shown in Figure 6. As we know, once concrete is subjected to acid attack, the compressive strength of the concrete will be reduced gradually [24]. The exposure times of 300 and 510 d did not exert any remarkable differences on concrete strength, and the different locations in the cooling tower also had no obvious influences on concrete strength (Figure 6). This result suggests that the concrete inside the tower shows no signs of corrosion.

**Figure 7:**
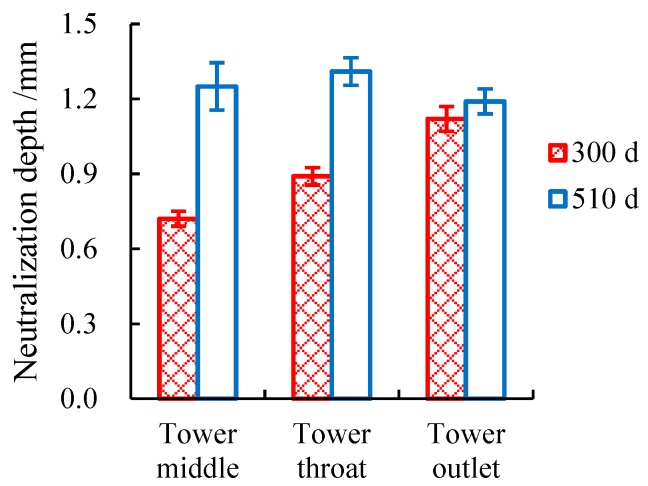
Neutralization depths of in situ specimens at different locations.

The neutralization depths of the concrete specimens were determined using a 1% phenolphthalein ethyl alcohol indicator, as shown in Figure 7. The neutralization depths of the in situ specimens were all small. Even after 510 d of exposure in the cooling tower with flue gas injection, the highest value of neutralization depth was only approximately 1.2 mm, and no remarkable difference was observed among the specimens at different locations. The reason for this is that the concrete specimens did not suffer from a strong acid attack, and the high relative humidity in the cooling tower caused a small carbonation depth. This result is consistent with those for the measured acid gas concentration and condensate pH values.

The monitoring results of flue gas and the durability performance of the in situ concrete specimens show that the injection of the cleaned flue gas into the cooling tower did not evidently increase the corrosion risk in the cooling tower concrete. As a result, heavy-duty anti-corrosion protection or specially designed concrete is not necessary. Such findings are of great significance for saving costs in the construction and maintenance of a natural draft cooling tower with flue gas injection.

## 4. Numerical Simulation of Flue Gas Concentration in the Cooling Tower

Theoretically, the flue gas of coal-fired plants, even after desulfurization treatment, still contains low concentrations of acidic gas and at least high concentrations of CO_2_ [2,3]. Why is there almost no acidic SO_2_ gas nor a high concentration of CO_2_ in the monitored data of this study? To answer this question, a finite element method was used to simulate the flow and diffusion of the cleaned flue gas in the cooling tower and to obtain its concentration distribution.

### 4.1. Finite Element Model

**Figure 8:**
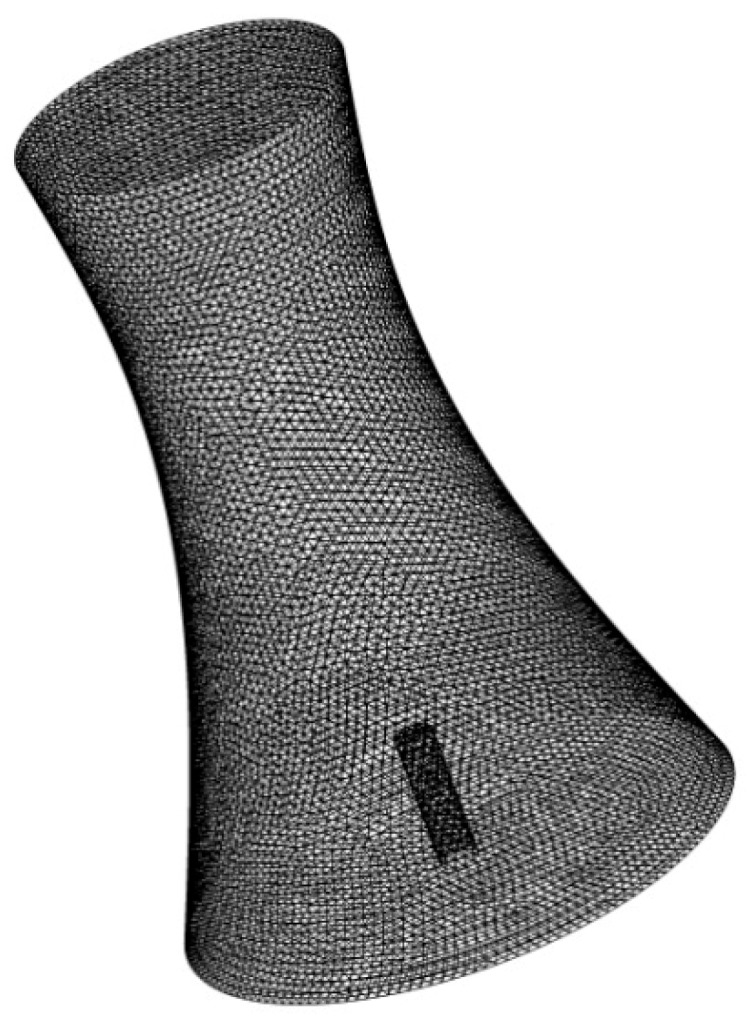
Finite element model of the cooling tower with a flue gas tube.

Fluent is a commonly used software for the simulation of heat and mass transfer in a cooling tower [13,25]. The cooling tower in this study was initially modeled using the Gambit pre-process software, and then imported into the Fluent software. The height of the tower was 150 m, and the diameters of the base bottom, outlet, and flue gas tube were 100, 60, and 7 m, respectively. The flue gas outlet and air inlet at the bottom of the cooling tower were set as velocity inlets, and the tower outlet was set as a free outlet. For simulation convenience, the original horizontal flue gas pipe was replaced with a vertical flue gas tube at the central position, and the elevation of the flue gas outlet remained the same, at 41 m. The detailed finite element model of the cooling tower with a flue gas tube is plotted in Figure 8, which is composed of 756,892 units, 1,538,959 faces, and 139,234 nodes.

According to the original data of the cleaned flue gas provided by Lu’an Power Station, the initial temperature and speed of the flue gas were 48 °C and 15 m/s, respectively. The major composition of the cleaned flue gas is shown in Table 5. In consideration of the fluctuation in air temperature inside the tower in different seasons, the mean monthly temperature of 20.12 °C in April in spring was set as the representative temperature of the air inside the tower.

To simulate the concentration diffusion of the flue gas in the cooling tower, which belongs to a transportation process of chemical components, the turbulence model of the k-ε double equation and the species transport model were used [26,27]. In addition, a segregated implicit solver was utilized, and the effects of energy exchange and gravity were considered in the simulation to make the results approximate reality.

### 4.2. Results of Numerical Simulation

**Figure 9:**
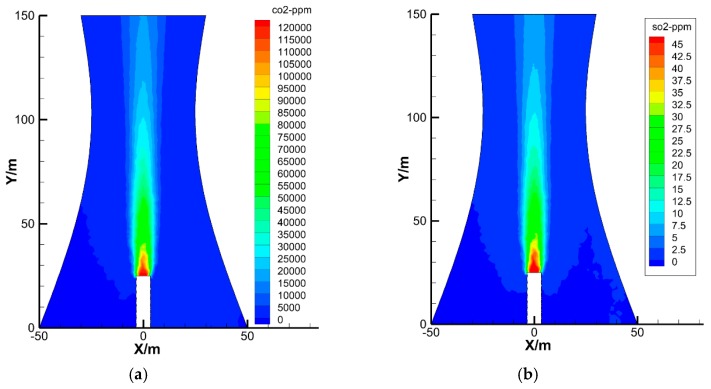
Concentration nephograms of CO_2_ and SO_2_ in the cooling tower. (**a**) CO_2_; (**b**) SO_2_.

Figure 9 shows concentration nephograms of CO_2_ and SO_2_ in the cooling tower. The concentration distributions of CO_2_ and SO_2_ were similar and imitated a burning candle’s flame, but they had different concentration values. Generally, the concentration of CO_2_ or SO_2_ gas at the central point of the tower was the highest, and the concentration decreased gradually as CO_2_ or SO_2_ gas rose. The initial concentration of CO_2_ at the central point was approximately 1.2 × 10^5^ ppm. The value decreased to approximately 1.5 × 10^4^ ppm at the tower outlet.

**Figure 10:**
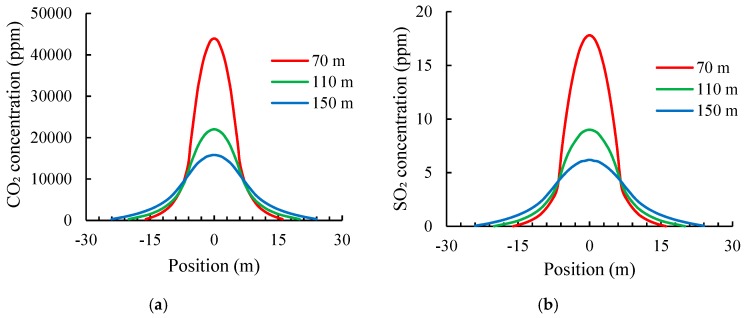
Concentration distributions of CO_2_ and SO_2_ in the cooling tower. (**a**) CO_2_; (**b**) SO_2_.

Three horizontal positions in the tower middle (70 m), tower throat (110 m), and tower outlet (150 m) were selected to further explore the vertical and radial distributions of flue gas concentration in the cooling tower. The concentration distributions of CO_2_ and SO_2_ along the horizontal direction are plotted in Figure 10.

The horizontal distribution curves of CO_2_ or SO_2_ concentration in the cooling tower were similar to a typical normal distribution curve that exhibits characteristics of a high middle part, low two sides, and symmetric left and right sides. The concentration of CO_2_ not only decreased as the flue gas rose but also decreased quickly along the horizontal direction. Particularly, at the height of 70 m, the concentration of CO_2_ at the central point was approximately 4.34 × 10^4^ ppm, whereas the concentration decreased to close to the concentration of CO_2_ in natural atmosphere when it diffused to the diameter of 24 m. That is, the effective diffusion diameter of CO_2_ at this height was approximately 24 m. Similarly, the effective diffusion diameters of CO_2_ at heights of 110 and 150 m were about 28 and 40 m, respectively. These dimensions are all smaller than the corresponding diameter of the cooling tower. In other words, before the flue gas reached the inner surface of the cooling tower, its concentration of CO_2_ decreased to near the concentration of the natural atmosphere. These findings agree well with the measured CO_2_ concentration near the inner surface of the cooling tower at different locations.

The concentration distribution of SO_2_ in the cooling tower was similar to that of CO_2_ but with a lower concentration. By further simulating the flue gas distribution in different seasons, we found that the concentration distribution of flue gas in the cooling tower was hardly affected by seasonal changes.

## 5. Discussion

### 5.1. Verification of Numerical Simulation Results

**Figure 11:**
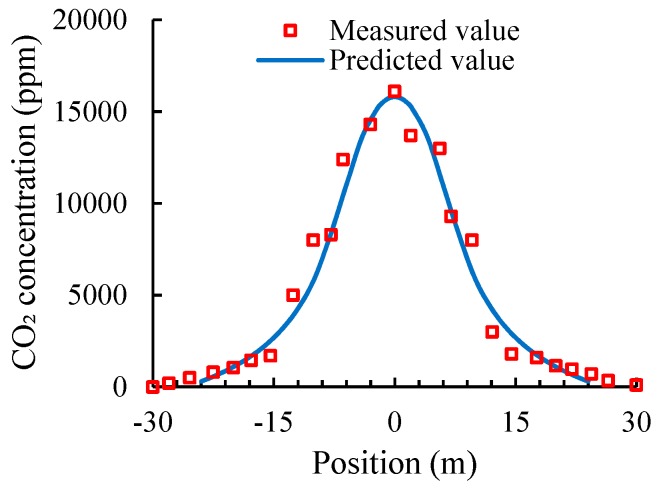
Comparison of CO_2_ concentrations measured in situ and in the numerical simulation.

Because it is difficult to measure the flue gas concentration inside the cooling tower, the CO_2_ concentrations along the radial direction at the tower outlet were measured to further verify the correctness of the numerical simulation results. Initially, within the range of 0–7.5 m from the edge of the tower, the concentration of CO_2_ was low, and the values were between 350 and 550 ppm, similar to the concentration of CO_2_ in external natural atmosphere. Gradually, within the range of 10–15 m from the tower edge, the CO_2_ concentration increased. When the distance from the tower edge exceeded 17.5 m, the concentration of CO_2_ increased rapidly. A comparison of the measured data of radial CO_2_ concentration at the tower outlet and those in the numerical simulation is plotted in Figure 11.

### 5.2. Concrete Corrosion Risk Caused by Flue Gas

The measured data were consistent with the results of the numerical simulation, thereby confirming the correctness of the numerical simulation of flue gas concentration distribution in the cooling tower. The CO_2_ concentration distribution in the cooling tower exhibited characteristics of a typical normal distribution. Even at the height of 150 m, the CO_2_ concentration at the tower center was still very high, with a value close to 16,000 ppm. This value is approximately 35 times that of the CO_2_ concentration in ambient natural atmosphere. However, due to the normal distribution characteristics of the flue gas concentration in the cooling tower, the concentration decreased rapidly as the flue gas diffused to the edge of the cooling tower. The concentration of the flue gas almost dropped to the same level as that in ordinary atmosphere before reaching the tower wall due to the gigantic diameter of a normal cooling tower. Therefore, we conclude that cleaned flue gas discharged from the flue gas tube into the cooling tower does not evidently increase the corrosion risk in the concrete tower wall.

The cooling towers in this project have been operating for five years since their completion. The tower concrete still shows no signs of corrosion, which is consistent with the analysis results in this study. The savings in expensive anti-corrosive coatings and specially designed concrete can result in significant cost savings for the construction of cooling towers with flue gas injection.

## 6. Conclusions

The following conclusions were derived from the field monitoring, experimental investigations, and numerical simulation in this study.

1. The flue gas conditions near the inner surface of a cooling tower are remarkably different from those in the tower center. The flue gas in the tower center has a high concentration of acid gas, whereas that near the inner wall of the cooling tower has a low concentration of acid gas and has almost no corrosion risk.
2. The condensate attached to the inner wall of the cooling tower is slightly acidic, with a pH value ranging from 6.0 to 6.5. It has low contents of corrosive media. The macromorphology, compressive strength, and neutralization depth of the in situ concrete specimens indicate that the condensate attached on the inner wall of the cooling tower poses no strong corrosive risk to the tower concrete.
3. The finite element model established in this study can effectively simulate and predict the concentration distribution of flue gas in a cooling tower. The predicted results are consistent with the measured data. Generally, the concentration of flue gas at the tower center is the highest and decreases rapidly as it leaves the center. Given the gigantic diameter of a cooling tower, the flue gas is not expected to cause a high corrosion risk to the tower concrete.

## Figures and Tables

**Table 1:** Concrete mixture proportion (kg/m^3^).

	Cement	Sand	Stone	Water	Water Reducer	Fly Ash	Silica Fume
Dosage	378.7	701.3	1046.7	180	7.6	70.7	23.6

**Table 2:** Chemical compositions of cement, fly ash, and silica fume (wt.%).

Item	SiO_2_	Fe_2_O_3_	Al_2_O_3_	CaO	MgO	Ignition Loss
Cement	22.3	3.2	5.0	64.9	0.9	1.32
Fly ash	57.2	4.5	30.3	2.5	0.6	2.65
Silica fume	96.0	0.7	0.8	0.2	0.5	2.12

**Table 3:** Detailed monitoring plan.

Item	Location	Elevation (m)	Monitoring Contents
1	Tower middle	70	T, RH, CO_2_, SO_2_, condensate, and specimens
2	Tower throat	112	T, RH, CO_2_, SO_2_, condensate, and specimens
3	Tower outlet	150	T, RH, CO_2_, SO_2_, condensate, and specimens

**Table 4:** Average pH values and anion contents in the condensate at different locations (mg/L).

Item	Tower Middle	Tower Throat	Tower Outlet
pH value	6.45	6.06	6.16
F^−^ ion	1.9	2.1	3.7
Cl^−^ ion	17.2	70.2	29.2
SO_4_^2−^ ion	3.8	26.9	91.7
NO_3_^−^ ion	21.8	4.0	5.9

**Table 5:** Major composition of the cleaned flue gas (%).

Item	CO_2_	O_2_	N_2_	SO_2_	H_2_O
Volume fraction	11.76	5.64	71.22	0.0048	11.38
